# Functional Cognitive Disorder in a Kent Memory Service: A Six-Month Evaluation of Prevalence, Diagnostic Inconsistency, Cognitive Profile, and the Case for Operationalised Criteria.

**DOI:** 10.1192/bjo.2026.11627

**Published:** 2026-06-30

**Authors:** Lucia Laskowski, Suresh Thapaliya, Madhusudan Dalvi, Rafey Faruqui, Julie Chundamala

**Affiliations:** 1 Kent and Medway Mental Health Trust, Canterbury, United Kingdom; 2 Kent and Medway Mental Health Trust, Folkestone, United Kingdom; 3 Kings College London, London, United Kingdom; 4 University of Kent, Canterbury, United Kingdom

## Abstract

**Aims:**

Functional Cognitive Disorder (FCD) is increasingly recognised within memory services, yet its non-organic symptom profile, subjective–objective mismatch, and inconsistent cognitive performance continue to pose diagnostic challenges. Although conceptual frameworks, such as those proposed by Harriet Ball et al. (2020), have advanced understanding of FCD, translation into routine clinical practice remains variable.

This six-month retrospective service evaluation examined the prevalence, clinical characteristics, and diagnostic pathways of FCD within the South Kent Coast Memory Assessment Service. The project assessed diagnostic reach, cognitive profiles, psychiatric comorbidity, and service timelines while evaluating the practical utility and limitations of current diagnostic frameworks.

**Methods:**

A total of 550 consecutive referrals (October 2024–March 2025) were reviewed. Patients were evaluated against six operationalised criteria grounded in established FCD models: subjective cognitive concerns, objective–subjective mismatch, internal inconsistency, absence of neurodegenerative disease, psychological contributors, and functional impact. Demographic data, cognitive assessments (ACE-III, MMSE), neuroimaging, psychiatric comorbidity, and service outcomes were analysed descriptively.

**Results:**

Thirty individuals (5.4%) met operational criteria for FCD or FCD-like presentations, consistent with UK prevalence estimates. However, only seven received a formal FCD diagnosis; the remaining 23 were coded as Mild Cognitive Impairment or given non-specific labels despite exhibiting clear functional features, highlighting significant diagnostic drift. The cohort was predominantly female (60%), with a mean age of 67 years and high psychiatric comorbidity (77%).

Cognitive profiles showed preserved attention, language, and visuospatial abilities, with variable memory performance and disproportionately reduced phonemic fluency. Relatively intact encoding but impaired spontaneous recall, with strong cue dependence, supported positive diagnostic features of a functional cognitive profile. Neuroimaging revealed no progressive pathology.

The service pathway analysis revealed significant variability, with average durations from referral to assessment and referral to discharge being 138 and 156 days, respectively.

**Conclusion:**

These findings reinforce FCD as a prevalent and clinically significant presentation characterised by preserved cognitive function disrupted by anxiety-related performance interference. They also highlight limitations within the Harriet Ball et al. framework. While conceptually valuable, the criteria depend heavily on clinician interpretation of constructs such as “internal inconsistency” and “distress,” which lack clear operational thresholds and may contribute to the variability observed in diagnoses. Additionally, documentation gaps and inconsistencies in coding suggest that the current criteria are insufficiently structured for routine clinical practice.

A shift toward positively framed, operationalised diagnostic criteria, enhanced clinician training, and integrated psychological pathways is urgently required to improve diagnostic accuracy, reduce uncertainty, and optimise care for individuals with FCD.

